# Communication in health work during the COVID-19 pandemic

**DOI:** 10.17533/udea.iee.v38n3e09

**Published:** 2020-11-11

**Authors:** Maria Eunice Nogueira Galeno Rodrigues, Adriano da Costa Belarmino, Lívia Lopes Custódio, Ilvana Lima Verde Gomes, Antonio Rodrigues

**Affiliations:** RN, Master's student. Email: eunicegaleno@hotmail.com eunicegaleno@hotmail.com; 2 RN, Master's student. Email: adrian_belarmin@hotmail.com adrian_belarmin@hotmail.com; 3 Psychologist, Doctoral student Email: liviacustodio@yahoo.com.br liviacustodio@yahoo.com.br; 4 RN, Ph.D. Professor. Email: ilverde@gmail.com ilverde@gmail.com; 5 RN, Ph.D. Professor. Email: arodrigues.junior@uece.br arodrigues.junior@uece.br; 6 Graduate Program in Collective Health, State University of Ceará - UECE. Fortaleza-CE, Brazil. Universidade Estadual do Ceará State University of Ceará - UECE Fortaleza Brazil

**Keywords:** Coronavirus infections, health communication, patient care team, nursing, infecciones por coronavirus, comunicación en salud, grupo de atención al paciente, enfermería, infecções por coronavírus, comunicação em saúde, equipe de assistência ao paciente, enfermagem

## Abstract

**Objective.:**

Report on communication and qualified listening in nursing work in the face of the COVID-19 pandemic.

**Methods.:**

This descriptive, theoretical and reflexive report was developed by nurses between March 20th and May 25th 2020 at Emergency Care Services in the city of Fortaleza, Ceará, Brazil. Health communication served as the theoretical background for this research.

**Results.:**

Two main thematic categories were highlighted: (i) Resignifications of communication in the work relationships of the health team and (ii) Guided listening to users by nurses at the Emergency Care Services during the pandemic.

**Conclusion.:**

The experience revealed an excerpt of what is found under the conditions of the current situation resulting from COVID-19. Communication turned into an essential tool to maintain professional relationships and culminate in collaboration and cooperation of the team in order to provide a close relationship with the user and promote the quality of health care processes.

## Resumen

**Objetivo.:**

Relatar la comunicación y escucha calificada en el trabajo de enfermería frente a la pandemia por la COVID-19.

**Métodos.:**

Se trata de un relato descriptivo, teórico y reflexivo elaborado por enfermeros, del 20 de marzo al 25 de mayo de 2020, en Unidades de Atención de Emergencia de la ciudad de Fortaleza, Ceará, Brasil. La investigación posee fundamentación teórica en comunicación sanitaria.

**Resultados.:**

Se destacaron dos categorías temáticas principales: (i) Resignificaciones de la comunicación en las relaciones de trabajo del equipo de salud y (ii) la escucha dirigida de los usuarios por parte de las enfermeras de la UPAS durante la pandemia.

**Conclusión.:**

La experiencia vivida permitió visualizar una fotografía de la situación actual derivada de la COVID-19. La comunicación se convirtió en una herramienta fundamental para mantener las relaciones profesionales y facilitó la colaboración y cooperación en equipo para brindar una relación cercana con el usuario y promover la calidad de los procesos de atención y asistencia en salud.

## Resumo

**Objetivo.:**

Relatar a comunicação e escuta qualificada no trabalho da enfermagem diante da pandemia de COVID-19.

**Métodos.:**

Trata-se de um relato descritivo, teórico e reflexivo realizado por enfermeiros no período de 20 de março a 25 de maio de 2020 em Unidades de Pronto Atendimento da cidade de Fortaleza, Ceará, Brasil. A pesquisa possui fundamentação teórica na comunicação em saúde.

**Resultados.:**

Destacou-se duas principais categorias temáticas: (i) Ressignificações da comunicação nas relações de trabalho da equipe de saúde e (ii) A escuta direcionada dos usuários pelos enfermeiros das UPAS na pandemia.

**Conclusão.:**

A experiência vivenciada possibilitou visualizar um recorte do que se encontra sob as condições do atual quadro decorrente do COVID-19. A comunicação tornou-se ferramenta imprescindível para manter relações profissionais e culminar em colaboração e cooperação da equipe de maneira a proporcionar estreita relação com usuário e promoção da qualidade dos processos de cuidado e assistenciais de saúde.

## Introduction

Nursing goes through periods of change in the global scenario with the coping of the pandemic of COVID-19. Unexpectedly, there are changes in the routine of health services, organizational structures and professional relationships. At this moment, health teams, particularly nursing, are moving in the change and adaptation process, as well as their relationship with light technologies.(1,2) Conceptually, these are relationship technologies, such as welcoming, bonding, independence, accountability, and management as a way of managing work processes.(3) The nurse, as a manager of the nursing team, has the autonomy to occupy all the spaces within their range, whether involving users or professionals, consciously and targeting the subjects' specific needs, aiming for the humanization of health care processes.(4)

From the perspective of the organization of health care actions, the health authorities, surveillance entities and the scientific society established flows in intermediate-complexity care for people experiencing health problems due to COVID-19 to use health services, including Emergency Care Services (UPA). Within this logic, the UPA is important in this process because it is a secondary-level health institution that acts as a gateway, attends to the population in emergency situations, identifying patients with symptoms of COVID-19, and uses care protocols to provide care in the prevention, maintenance and recovery of users seeking the health system.(5)

Thus, light technologies such as communication are fundamental in confronting the pandemic, in performing humanized practices at times of social and health crisis, focused on the desires and needs of the population. Therefore, the relationship between the professional and the user will be a benevolent relationship. The usability of light technologies and humanized care are important in this process.(6) Understanding communication as a light technology that modifies the work processes in the UPA within the pandemic context can represent a model of health team organization and cooperation to improve the quality of care for patients. Therefore, there is a need for studies focusing on the perspective of communication in health work, especially in teams that work in a situation of constant pressure, such as emergency care.

In a critical context such as the COVID-19 pandemic, the study contributes to discuss the interaction of the health team and work models in an emergency, clinical and surgical environment in which communication and listening is a key element to achieve quality goals in health and reduce risks and mortality. In that sense, the objective of this study is to report on communication and qualified listening in nursing work in view of the Covid-19 pandemic.

## Methods

This is a descriptive, theoretical and reflexive experience report. These reports favor the production of new knowledge through the construction process, turns experience into a theoretical interpretative object of study and promotes the recovery of phenomena in an orderly manner.(7) The proposal is to discuss the activities developed by two emergency nursing professionals working in UPAs located in Fortaleza, Ceará, during the COVID-19 pandemic, carried out by the authors with a determined and known research objective and justifications for the development of the study. The state was one of the main epicenters in the Northeast during the pandemic peak, with 188,451 confirmed cases and 8,010 deaths by August 8, 2020.(8)

The UPAs are dynamic spaces of pre-hospital emergency care, located in targeted regions of the city where the study was carried out. As parts of the Brazilian Emergency Care Network (RUE), they are places of organization and operation of complex secondary healthcare flows.(9) The care processes to compose this experience report took place between March 20 and May 25, 2020. The choice of the UPA as the place of study for the main research objective was intentional. To define the themes in the report, an adaptation of Minayo's thematic analysis was applied, using the most relevant words, expressions, and themes, built with the reporters' experience, based on the material of the results transcribed in Microsoft Word.(10)

The Consolidated criteria for reporting qualitative studies (COREQ) were used to develop the research: 32- *item checklist* for methodological consistency and quality of the article.(11) In this context, the experience described was discussed from the perspective of the Interprofessional communication process, interpreting this instrument in health work as a phenomenon with complex influences in the interprofessional relationships of the health team. It involves one of the aspects of interprofessional practice to overcome fragmentation in care and achieve problem-solving ability with quality and integrality.(12)

## Results

In the analysis of the reports during the period, two main thematic categories were highlighted: resignifications of communication in the work relationships of the health team and the guided listening to users by nurses from the UPAs during the pandemic.

### Resignifications in the work relationships of the health team

Initially, the care flows of the UPA were defined to identify symptomatic users with signs and symptoms of COVID-19, such as cough, fever, muscle pain, headache, respiratory distress and other components of the flu syndrome and/or acute respiratory distress syndrome, which guided the lines of care. Patients with suspicion and mild symptoms (oxygen saturation -SatO2 >92%) were evaluated by the physician in the office, instruction was provided and the patients were released for social distancing at home. Cases considered severe with hyposaturation (SatO2<92%) were hospitalized to start oxygen therapy, receive confirmatory tests, receive medication, and cases with severe pulmonary impairment and risk of respiratory arrest were submitted to orotracheal intubation and protective mechanical ventilation.

Developing nursing care activities in this dynamic represented restructuring and resignifying communication between health team professionals to achieve effective and quality care. The communication process in these professional relationships suffered from the precarious work imposed by the pandemic though, and therefore had to be modified. Seeking to resolve communication deficits that could culminate in quality losses in guided care, meetings and training were held to align the health team's care actions. In this period, the dynamics of the emergency area increased significantly and measures had to be aligned through communication, mainly between nurses and physicians during the visits due to the demand, the risks of mortality in severe cases, and the overcrowding of the sectors due to the delay in transfers.

The communication process contributes, however, for the cooperation and collaboration to culminate in the development of integrated intra-team practices, in decision making on the applicability of care to achieve the patient's wellbeing. In addition, this tool improves the relationship between nurse and physician, promoting interrelations and harmony in health work processes.

In this context, communication turned into an essential tool to maintain interprofessional relationships and culminate in collaborative actions in the team. The difference that allows quality communication processes in the health team and the noises that disrupt these relationships proved to be tenuous. One example is rapid orotracheal intubation and its systematization as a health procedure. This involves sedoanalgesia, introduction of the tube with a guidewire, removal of the wire while maintaining the plunger, clamping of the tube with tweezers, installation of the ventilator, and initiation of mechanical ventilation, being considered a complex procedure in which any failure in the communication of the medical team with the nursing team culminates in risks for the patient.

In this premise, communication was also the basis for decision making and for the development of integrated practices among health team professionals in the care and treatment processes directed to contaminated patients. Deciding on the correct time to, for example, change the nasal catheter to a non-rebreather mask and subsequently orotracheal intubation based on X-ray findings, gasometrical and clinical results literally meant the life and death of patients. These are complex decisions driven by evidence that was and is still under construction in the scientific literature. In most cases, the users' wellbeing was achieved successfully, either in restoring health or, in cases in which this was not possible, providing measures of comfort in terminal cases. It is important to emphasize that achieving harmony in communication processes and relationships among all health team professionals is not always satisfactory but proved to be the best route to quality care during the pandemic.

### Guided listening to the users by nurses at the UPA during the pandemic

The nursing team represents the health professionals who spend most of the time together with patients in all care processes. In the pandemic context, this was even more present due to the greater risks of complications, stabilization and care, especially enhanced by the non-permission of companions due to the infectious-contagious risk. Listening to health demands in patients suspected of COVID-19 involved dialogue, understanding and interpretation of the reports, reflection and decision. Countless patients arrived with mild and severe respiratory symptoms and being able to identify signs indicating infection by the novel coronavirus in the reports was one of the main steps in the flows of care and guidance.

Nevertheless, it is important to highlight that qualified listening is also something instinctive, subjective, inherent to the human being, which refers to the humanization of care processes. It demands comprehensiveness in care, makes it possible to achieve health goals and reduce dissatisfaction, disrespect and negative perspectives with health care. The suffering the pandemic imposed further required listening to patients and promoting reflexive health dialogue that culminates in care. In addition to guiding care by listening to health demands, it was also possible to act in the perspective of providing psychological and family comfort through professional contact with family members and patients.

The use of telephone calls and videoconferencing to have contact with family members showed that listening not only involves care through technical-medical procedures, but also promotes psychological comfort measures. Moreover, the perspective of listening represents a resource to promote mental wellbeing, relaxation, comfort, satisfaction.

In short, conducting guiding listening involves both solving basic health demands and priority and complex care. This complexity in each case, such as stabilization and clinical preservation of the patient and the promotion of physical and psychological well-being can be determined through appropriate listening to the user suspected of COVID-19, as a participant in the care process.

Thus, guided listening to the patient as an aspect that optimizes the care processes was considered to initially prioritize health care in the preservation of life and hemodynamic stabilization. These ranged from oxygen therapy to reduce hyposaturation to critical care in severe cases. In the listening processes by the team, interaction was essential, in which the presence of nurses, physicians and social assistants was necessary, whether in basic and complex care or in social and family relationship procedures and demands. It is challenging to achieve the structuring of the health team in the face of the pandemic, but intensifying the professional relationships and strengthening the health team work proved to be satisfactory to achieve positive results and reduce the impacts of the pandemic.

## Discussion

Coping with COVID-19 represents one of the greatest challenges in public health worldwide due to its high rates of illness and mortality, especially in socio-economically unfavorable contexts, such as Brazil, as well as in health work processes and relationships.(13,14) From the perspective of nursing, it is no different, being one of the most affected professions and with higher rates of morbidity, mortality, and absence from work during the pandemic.(15) In this context, the UPAs, together with other emergency care network equipment, represent the gateway for patients suspected of COVID-19 and complex relationships between health team professionals are developed in emergency care practices.(9)

In this premise, communication is an important element for integrated health practices to take place.(16) The disruption promoted by the need for distancing and the isolation the coronavirus imposes strengthens the construction of new forms of relationship, the realignment of care lines and practices.(17) As a member of the health team, the nurse represents one of the main care-related professionals involved in the dynamics of health work. Characteristically, the humanization models of health, the expanded clinic, changes in management and decision-making processes are theoretical and practical mechanisms addressed in the National Humanization Policy, in which light technologies such as communication are one of its main components.(18)

In this context, Merhy reflects in his studies the modes of health production within the work organization through the use of light, light-hard, and hard technologies in the development of living work.(19) In this perspective, hard technologies represent equipment and machines in which work is integrated; light-hard technologies are the professional knowledge, own knowledge and personal experiences that structure and organize work.(18) Differently, light technologies emphasize the professional and patient relationship, addressing subject-centered listening and the satisfaction of the needs of well-being, care, and health, affecting the care practices and the qualification of health work. They involve speaking, listening, interpreting, welcoming, bonding, representing, new knowledge, among others.(18,19)

In the pandemic, the communication mechanism represented a factor intrinsically related to the interaction processes, of cooperation between the health team involved, to improve the care developed, guaranteeing constitutional rights and integral and universal health services. First, the skills of proper listening to symptoms, screening, and determination of the severity and complexity of suspected cases determined the first line of care measures.(2) Communication, through qualified listening, is a vital health work instrument for interaction and cooperation of the intra-health team, with patients and family. It constitutes a tool to humanize the work processes and insert the subjective factor into the organizational dynamics as a catalyst for changes in health care.(20) It is important to emphasize, however, that the communication built with patients, family, and health team in the face of the pandemic is different.

The fear of infection by COVID-19 and its consequences determined different modes of communication defined by distancing and isolation.(17) Contacts made by telephone or videoconferencing with family members for patients with mild cases were strategies implemented to reduce fear, promote psychological well-being, and humanize care processes. Studies have been developed that emphasize digital tools such as videoconferencing for support during the COVID-19 pandemic.(21,22) A study on treatment for adolescents with eating disorders with family support found in videoconferencing an essential tool during the pandemic, although telehealth remains a challenge in access and resolutions.(22)

The use of digital technologies to reach family members, guide conducts, and improve patient well-being demonstrate a positive role during the pandemic, and investing in this perspective as an allied instrument in health care processes represents improvements in the subjective aspects of health work. Challenges are shown daily in the provision of care during the pandemic period and light technologies are revealed as mechanisms that can resolve difficulties during the development of the work and promote communication and listening aimed at solving problems and achieving interprofessional goals.

The use of communication in the Interprofessional team contributes to dynamic, engaging relationships, with effective and high-quality results. They involve cooperation, collaboration, leadership, and decision-making.(23,24) A study on interprofessional communication in the emergency sector between residents, physicians, and other members of the health team showed barriers to achieving effective communication, such as personal factors (fear, self-confidence), clinical environment (work overload, rapid changes in health teams), and lack of training.(24) In this context, the use of communicative processes to achieve better practices and care, in the interprofessional relationships of the team and in the quality of health care during the pandemic, is an undeniable tool in reporting, although its implementation, development, and qualification still needs improvements in the health system for the sake of better efficacy.(25)

The contributions are found in the identification of health team communication as a resource for improving interprofessional interactions, as well as assistance and care to patients in the pandemic context in emergency care institutions. The experience revealed an excerpt of what is found under the conditions of the current situation resulting from COVID-19. Communication has become an essential tool to maintain professional relationships and culminate in the team's collaboration and cooperation in order to provide a close relationship with the user. In addition, communicative processes such as guided listening to users and intra-team communication contribute to promote the quality of care and health care processes.

The main limitation identified in the report is the specific regional character of the health service and its specificities in the local context, but its results, such as communication to improve the relationship of the health team, can be replicated in countless contexts in the pandemic, thus becoming its main potential as well.

Nursing as a health profession, with its work processes directly affected by the pandemic, finds in light technologies such as communication and qualified and guided listening, strategies to improve care and relationships established with the health team, family and patient. This can solve risks, dissatisfaction, adverse events and morbidity and mortality of patients with COVID-19 in emergency care services.
